# Loss of Janus Associated Kinase 1 Alters Urothelial Cell Function and Facilitates the Development of Bladder Cancer

**DOI:** 10.3389/fimmu.2019.02065

**Published:** 2019-09-10

**Authors:** Vanessa Daza-Cajigal, Adriana S. Albuquerque, Joanna Pearson, Jennifer Hinley, Andrew S. Mason, Jens Stahlschmidt, Adrian J. Thrasher, Vibhash Mishra, Jennifer Southgate, Siobhan O. Burns

**Affiliations:** ^1^Institute of Immunity and Transplantation, University College London, London, United Kingdom; ^2^Department of Immunology, Royal Free London NHS Foundation Trust, London, United Kingdom; ^3^School of Medicine, Universidad Complutense, Madrid, Spain; ^4^Department of Immunology, Hospital Universitario Son Espases, Palma, Spain; ^5^Human Immunopathology Research Laboratory, Institut d'Investigació Sanitaria de Palma (IdISPa), Palma, Spain; ^6^Jack Birch Unit, Department of Biology, York Biomedical Research Institute, University of York, York, United Kingdom; ^7^Department of Histopathology, St James's University Hospital, Leeds, United Kingdom; ^8^Great Ormond Hospital for Children NHS Foundation Trust, London, United Kingdom; ^9^Section of Molecular and Cellular Immunology, Institute of Child Health, University College London, London, United Kingdom; ^10^Department of Urology, Royal Free London NHS Foundation Trust, London, United Kingdom

**Keywords:** bladder cancer, IFNγ signaling, immunodeficiency, JAK1, urothelium

## Abstract

Inherited Primary Immunodeficiency (PID) disorders are associated with increased risk of malignancy that may relate to impaired antitumor immune responses or a direct role for PID germline mutations in tumorigenesis. We recently identified germline loss of function mutations in Janus Associated Kinase 1 (*JAK1)* causing primary immunodeficiency characterized by infections and associated with early onset, fatal high-grade bladder carcinoma. Somatic mutations in *JAK1*, required for immune cell signaling in response to interferon gamma (IFNγ), have been associated with several non-hematopoietic and hematopoietic cancer cell types but pathogenic mechanisms remain largely unexplored. Here we demonstrate that JAK1 is required for the intrinsic IFNγ response of urothelial cells impacting immunogenicity and cell survival. Specifically, JAK1-deficient urothelial cells showed reduced surface expression of major histocompatibility complex class II (MHC II), intercellular adhesion molecule-1 (ICAM-1) and programmed death-ligand-1 (PD-L1) after IFNγ stimulation and were resistant to IFNγ-induced apoptosis and lymphocyte-mediated killing. In addition, we identify a previously unknown role for IFNγ signaling in modulating urothelial differentiation. Together, our findings support a role for urothelial cell JAK1 in immune surveillance and development of bladder cancer. Our results have implications for patients with rare JAK1 PID and, more broadly, inform development of biomarker and targeted therapies for urothelial carcinoma.

## Introduction

Widespread availability of next generation sequencing (NGS) has transformed the field of cancer, identifying candidate genes to enhance our understanding of tumorigenesis and enable stratification of patients for specific therapies. In the field of immunology, NGS has identified new monogenic forms of primary immunodeficiencies (PID): inherited disorders predisposing to infection, autoimmunity and malignancy. Genes identified to cause PID overlap with cancer susceptibility genes, so that clarifying the pathogenesis of PID has impact for oncology. We recently identified a new PID associated with compound homozygous loss of function mutations in the gene coding the signaling protein Janus-Associated Kinase 1 (JAK1) resulting in partial JAK1 deficiency (1). The clinical phenotype was characterized by immunodeficiency plus aggressive urothelial carcinoma that was fatal in the third decade of life, suggesting that impaired JAK1 function may be a predisposing factor for urothelial carcinoma.

JAK1 belongs to a family of widely-expressed tyrosine kinases essential for signal transduction from multiple cytokine receptors through activators of transcription (STAT) proteins (2, 3). Individual family members (JAK1, JAK2, JAK3, and Tyrosine kinase 2) associate with selected receptors and have non-redundant roles in cell signaling. Multiple receptors utilize JAK1 for signal transduction including members of the IL-2 receptor family (IL-2R, IL-7R, IL-9R, and IL-15R), the IL-4 receptor family (IL-4R, IL-13R), the gp130 receptor family (IL-6R, IL-11R, LIF-R, OSM-R CT-1R, CNTF-R, NNT-1R/BSF- 3R, and Leptin-R), and class II cytokine receptors (type I IFN-R, type II IFN-R, IL-10R) (2). Elucidating the non-redundant roles of JAK1 in cell biology has been complicated by the fact that complete JAK1-deficiency results in perinatal lethality as a result of neurological defects in murine models (3) precluding detailed examination of immune competence or malignancy risk.

Activation of the JAK1-dependent interferon gamma (IFNγ) signaling pathway is known to have direct effects on tumor cells, impacting numerous cell programmes including growth, apoptosis, proliferation, differentiation and migration (4–8). In addition, IFNγ induces immunoregulatory functions in cancer cells such as cytokine production, antigen presentation by major histocompatibility complexes (MHC) (9), expression of adhesion molecules such as intercellular adhesion molecule-1 (ICAM-1) (10) and ligands for receptors of immune checkpoints such as programmed death 1/programmed death-ligand 1 (PD-1/PD-L1) (11). In mouse models, loss of IFNγ signaling accelerated tumor initiation and progression, demonstrating a direct impact of IFNγ signaling on tumorigenesis, the tumor microenvironment and metastatic dissemination (5, 12).

To date, bladder urothelial carcinoma has not been described as a feature of other loss of function JAK-STAT or IFNγ-related PID, although different types of tumors have been reported in these conditions, including disseminated cutaneous squamous cell carcinoma (13–16). Somatic mutations in *JAK1* have been identified in multiple tumor types including high-risk bladder cancer, endometrial, colorectal, stomach, and prostate carcinomas (17–19) supporting the idea that alterations in JAK1 signaling, whether through loss or gain of function, could play a role in the pathogenesis of some epithelial cancers. Somatic mutations predicted to cause loss of JAK1 function are associated with reduced expression of IFN-associated genes in different tumor types (19). Although the cellular mechanisms have not been fully elucidated, truncating somatic *JAK1* mutations in gynecological carcinomas reduced IFNγ-induced MHC class I expression at the tumor cell surface, which could reduce immune recognition and facilitate immune evasion (17).

Here we examine the role of JAK1 in human urothelial cells and demonstrate a requirement of JAK1 for multiple functions including cell survival and interaction with immune cells. We also identify a potential role for JAK1 in modulating urothelial differentiation phenotype through IFNγ-signaling. Our findings provide new insight into immune and intrinsic signaling regulation of urothelial cells and support a role for JAK1 in the pathogenesis of bladder cancer.

## Materials and Methods

### The Cancer Genome Atlas Analysis

Somatic variants for the muscle invasive bladder cancer (BLCA) cohort from The Cancer Genome Atlas (TCGA) were accessed from the Genome Data Commons (20) as part of dbGaP project 19625. Variants which overlapped the predominant JAK1 transcript (ENST00000342505.5) were extracted and their impact assessed using the Ensembl Variant Effect Predictor (21) including SIFT 4G (22) and Poly-Phen 2 (23), with additional analysis of deleterious effects using HMMvar v1.1.0 (24). Mutations and their effects were presented against the JAK1 protein sequence (InterPro P23458).

### Immortalized and Normal Human Urothelial Cell Cultures

An immortalized normal human urothelial (NHU) cell subline produced by retroviral transduction with human telomerase reverse transcriptase (hTERT) cells as detailed elsewhere (25), was used in this study. The subline, referred to as Y235hTERT, was previously characterized at passage 40 against the pre-immortalized parental line (passage 7) using comparative genomic hybridization. The Y235hTERT cell lines for this study were cultured using protocols detailed in full elsewhere (26) and used within 20 passages of the CGH analysis. JAK1 knock down (KD) and scrambled control (Sc) hTERT urothelial sub-lines were generated using lentiviral vectors expressing short hairpin RNA (shRNA) sequences and utilized for all experiments unless otherwise indicated. shRNA technology permitted generation of cell lines with sub-total deficiency similar to the effect of loss of function mutations seen in our patient (1).

For differentiation studies only, normal human urothelial (NHU) cells obtained ethically with appropriate informed consent and Research Ethics Committee approvals were maintained *in vitro* as non-immortalized (finite) cell cultures. For routine culture, NHU cells were grown as adherent monolayers on Primaria™ plasticware (BD Biosciences) in low calcium (0.09 mM) keratinocyte serum-free medium (KSFM) containing bovine pituitary extract and recombinant epidermal growth factor (Life Technologies) supplemented with 30 ng/ml cholera toxin (KSFMc). NHU cells were sub-cultured by trypsinization at just-confluence and used in experiments between passages 3–5. Differentiation experiments described here were performed on five independent NHU cell cultures from five different donors. Due to the finite nature of these lines, no genotyping of individual cell cultures was performed. Differentiation of NHU cells was induced in just-confluent cell cultures using 1 μM troglitazone (TZ) as peroxisome proliferator-activated receptor-gamma (PPARγ) activating ligand with concurrent 1 μM PD153035 to block epidermal growth factor receptor (EGFR) activation, as previously described (27).

All Y235hTERT cell lines and NHU cell cultures were tested regularly for contamination by *Mycoplasma* spp. using polymerase chain reaction-based kits and DNA-intercalating fluorescent stains for presence of extranuclear DNA. For all stimulation experiments described, concentrations of IFNγ were optimized for each experiment.

### Lentivirus Preparation and Transductions

pGIPZ vectors carrying the short hairpin RNA against JAK1(TAGTACACACATTTCCATG) or scrambled control (TGAACTCATTTTTCTGCTC) sequences as well as puromycin resistance cassette and turbo-GFP marker for selection were supplied by University College London Open Biosystems (London UK). Lentivirus stocks were prepared by transfection of 293T cells (80–90% confluence) cultured in DMEM medium and 10% heat-inactivated fetal bovine serum, with the envelope plasmid 17.5 μg pMD.G2 (VSV-G/envelop), 32.5 μg p8.74 plasmid (gag-pol) and 25 μg vector construct with the transfection reagent PEI/Opti-MEM™ following the manufacturer's instructions (Promega). Medium was replaced 5 h post-transfection and medium was harvested after 24 and 48 h, cleared by centrifugation (4,000 rpm, 5 min), filtered through 0.22 μm filters and left to spin for 2 h 4°C 50,000 g. Viruses were titrated on 293T cells by scoring GFP-positive cells (flow cytometry) 3 days post-transduction. Virus stocks were stored at −80°C. Transductions of urothelial cells were carried out by infection at a multiplicity of infection of 1:10 for 6 h, before replacing virus-containing medium with fresh medium. Cells were selected in puromycin-containing medium and the efficiency of transduction was assessed by percentage of GFP-positive cells. Loss of JAK1 expression in JAK1-deficient hTERT urothelial cells was verified by RT-PCR.

### Determination of mRNA Levels by Real Time-Quantitative Polymerase Chain Reaction (RT-qPCR) and Reverse Transcription Polymerase Chain Reaction (RT-PCR)

Cells were left unstimulated or stimulated with 1 ng/ml of IFNγ for different time points. Total RNA from hTERT urothelial cells was extracted using RNAeasy kit (Qiagen). RNA from NHU cells was extracted in TRIzol® reagent (Thermo Fisher Scientific, Loughborough, UK); any contaminating DNA was digested using a DNA-free kit (Thermo Fisher Scientific). RNAs were converted to cDNA by reverse-transcription using QuantiTect reverse transcription kit (Qiagen).

Determination of mRNA abundance was performed by RT-PCR using specific primers (Table S1) and QuantiTect SYBR® Green PCR Kit (Qiagen) according to manufacturer's instructions. Fold changes were calculated using the DDCT2 [–Delta Delta C(T)] method and results were normalized with respect to the values obtained for the endogenous GAPDH cDNA.

### Flow Cytometry Analysis

Sc and KDhTERT urothelial cells, ± addition of 5 ng/ml of IFNγ for different time points, were detached using Accutase® solution (A6964, Sigma Aldrich), labeled with fluorescent-conjugated antibodies (see Table S2) and washed with phosphate buffered saline (PBS). For STAT1 phosphorylation analysis, cells ±1 ng/ml IFNγ for 10 min were fixed and permeabilized using fix buffer I and Perm Buffer III (BD Biosciences) for 30 min at 4°C, washed with PBS and labeled with 5 μL anti-pSTAT1 antibody (BD Biosciences) for 60 min in the dark. For all flow cytometry (BD LSRFortessa) experiments 10,000–30,000 gated events were collected and analyzed using FlowJo software.

### Cell Viability and Apoptosis Assays

Sc and KD hTERT urothelial cells were stimulated with IFNγ using different time points and concentrations. Alamar Blue® (AB), diluted 1:10 with KSFMc, was added to urothelial cells grown in 96-well plates (4,000 cells/well). After 3 h incubation at 37°C, absorbance was measured at 560 and 620 nm. AB reduction was calculated according to manufacturer's instructions (AbD Serotec, Kidlington, UK). Apoptosis was determined by flow cytometry using APC Annexin V apoptosis detection kit with Propidium Iodide (PI) according to manufacturer's instructions (BioLegend 640932).

### Lymphocyte Cytotoxicity Assay

Healthy donor peripheral blood mononuclear cells (PBMCs) were isolated by Ficoll™ gradient and frozen in 10% dimethyl sulfoxide (DMSO). To control for differences in the frequency of lymphocyte subsets between donors, PBMCs collected from a single donor buffy coat were stored cryopreserved and used for all killing assays shown. PBMC were thawed and re-suspended in RPMI (Invitrogen) with 10% heat-inactivated fetal bovine serum and monocytes removed by plastic adherence (1 h, 37°C). Urothelial cells were cultured in 96-well plates ± IFNγ (5 ng/ml) for 30 h and subsequently co-cultured overnight with interleukin-2 (IL-2) 25 U/ml (Roche) + monocyte-depleted PBMCs (50:1). Cells were detached using Accutase® solution, washed with PBS and resuspended in 200 μl DNA staining solution which specifically stains nuclei in dead cells and allows identification of necrotic cells (NKTEST, Glycotope Biotechnology). Urothelial cells were gated based on green fluorescent protein (GFP) expression and analyzed by flow cytometry.

### Immunohistochemistry

Five micrometer formalin-fixed tissue sections were dewaxed in xylene, then rehydrated through ethanol into water. Antibodies are detailed in Table S3. Following appropriate antigen retrieval (Table S3), sections were incubated with optimally titrated primary antibodies and immunodetectedusing secondary antibodies linked to streptavidin-biotin horseradish peroxidase complex (DAKO), ImPRESS™ Excel Polymer system (Vector labs), or BenchMark Ultra automated staining system (Ventana) (Table S3). All slides were counterstained in Mayer's haematoxylin and mounted in DPX (Sigma). Control slides and tissues were included to check specificity.

### Statistical Analysis

Statistical analysis was performed using Graphpad Prism 5.1 Software. Associations between JAK1-deficient and control cells were tested using one-way ANOVA and appropriate post-test, or a two-tailed Mann Whitney *U*-test. A *p* < 0.05 was considered significant.

## Results

### Partial JAK1 Deficiency Impairs STAT1 Phosphorylation and Expression of IFNγ-Inducible Genes in hTERT Urothelial Cells

**Figure 1:**
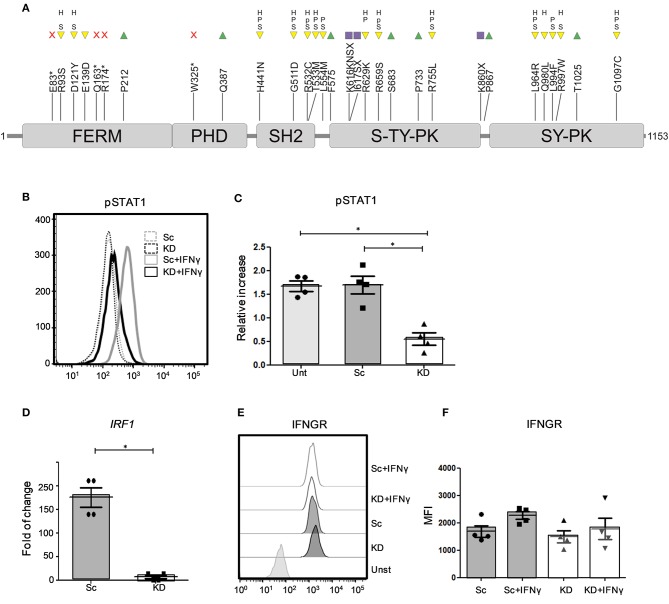
STAT1 phosphorylation and expression of *IRF1* mRNA is impaired in JAK1-deficient hTERT urothelial cells. **(A)** JAK1 protein (InterPro P23458) structure showing core domains, TCGA BLCA variants and their predicted impact. Protein domains: FERM (4.1 protein, ezrin, radizin, moesin domain), PHD (pleckstrin homology-like domain), SH2 (Src homology 2), S-TY-PK (unknown specificity serine-threonine/tyrosine protein kinase), SY-PK (serine-tyrosine protein kinase). Variants: non-sense (red cross), missense (yellow inverted triangle), synonymous (green triangle), frameshift (purple square). Variant effects: S (SIFT score < 0.05), P (Poly-Phen score > 0.908), p (Poly-Phen score > 0.446 & ≤ 0.908), H (HMMvar score > 2). A single 5′ UTR modifier mutation not shown. **(B,C)** Analysis of JAK/STAT signaling by flow cytometry in untransduced (Unt), Sc and KD hTERT immortalized urothelial cell lines after stimulation with IFNγ (1 ng/ml) for 24 h. Data **(A)** is from a representative experiment, data **(B)** is from three independent experiments. Two-tailed Mann Whitney test. **(D)** RTqPCR analysis of *IRF1* mRNA expression from KD and Sc hTERT immortalized urothelial cell lines after stimulation with IFNγ (1 ng/ml). Data is from four independent experiments. Two-tailed Mann Whitney test.^*^P < 0.05 Error bars represent the SE. **(E,F)** Flow cytometry analysis of IFNGR expression in KD and Sc hTERT immortalized urothelial cell lines following stimulation with IFNγ (5 ng/ml) for 2 days. The graph shows mean values ± SD. Data is from three independent experiments. One-way ANOVA with Tukey's Multiple Comparison Test.

While our patient remains the only reported individual with partial JAK1 deficiency caused by germline loss of function mutations, somatic variants in *JAK1* have been described in other patients with very high-risk bladder cancer (18). Analysis of the muscle invasive bladder cancer (BLCA) cohort from The Cancer Genome Atlas (TCGA) (20) identified 31 different *JAK1* variants in 25/412 TCGA BLCA samples, including single nucleotide variants predicted to alter JAK1 protein function and non-sense/frameshift variants predicted to impair JAK1 signaling (Figure 1A).

To model and test the impact of loss of JAK1 function in urothelial cells, we generated a JAK1 KD hTERT immortalized urothelial cell line using lentiviral vectors expressing shRNA sequences. Compared to control Sc shRNA, JAK1 shRNA substantially reduced *JAK1* mRNA expression (Figure S1A). To confirm functional knock down of JAK1, we studied JAK1-mediated activation of STAT1 proteins in response to IFNγ stimulation, using flow cytometry. We observed a significant decrease in STAT1 phosphorylation following IFNγ stimulation in the KD cell line compared to untransduced and Sc shRNA lines (*p* < 0.05) (Figures 1B,C and Figure S1B). The effect was similar to the reduced, but not abolished, STAT1 phosphorylation observed in fibroblasts of the patient with JAK1 deficiency following IFNγ stimulation (Figure S1C). Expression of mRNA for the interferon inducible transcription factor *IRF1* was also significantly lower in KD than Sc urothelial cell lines following stimulation with IFNγ, indicating impaired downstream gene regulation in JAK1 deficiency (Figure 1D). Reduced responses to IFNγ were not due to alteration in expression of the IFNγ receptor (IFNγR) as Sc and KD hTERT urothelial cells displayed comparable IFNγR surface expression both at baseline and following IFNγ stimulation (Figures 1E,F and Figure S1B).

### JAK1 Deficiency Alters MHC, ICAM-1, and PD-L1 Expression in hTERT Urothelial Cells

**Figure 2:**
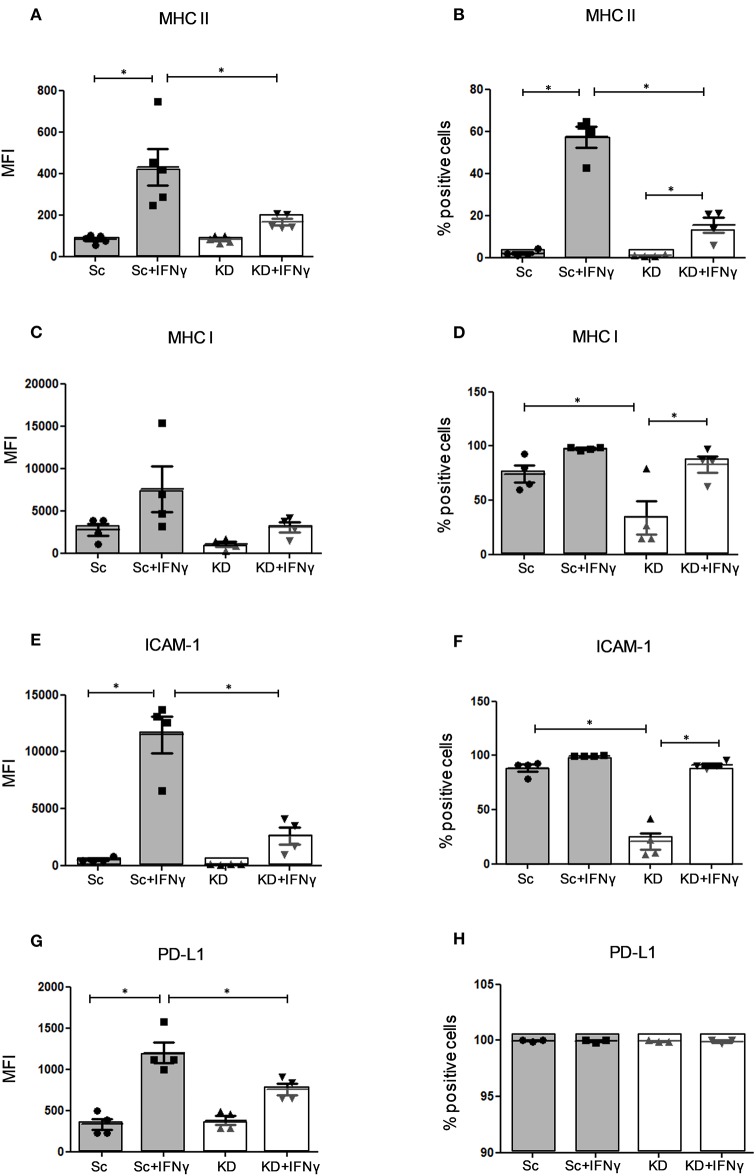
Analysis of MHC I/II, ICAM-1 and PD-L1 expression in hTERT urothelial cells by Flow Cytometry. Flow cytometry analysis of surface receptor expression in KD and Sc hTERT immortalized urothelial cell lines before and after IFNγ stimulation. Mean fluorescence intensity and percentage of positive cells are shown for **(A,B)** MHC II **(C,D)** MHC I **(E,F)** ICAM-1, and **(G,H)** PD-L1 expression. Data is from four independent experiments for **(A–F)** and from three independent experiments **(G,H)**. One-way ANOVA with Tukey's Multiple Comparison post-test. Graphs shows mean values ± SE and regulation of the expression compared to untreated. ^*^P < 0.05. Error bars represent the SE.

It was previously shown that IFNs regulate expression of MHC, ICAM-1 and PD-L1 in cancer cells (9–11). We examined the expression of these cell surface molecules in JAK1-deficient and Sc hTERT urothelial cell lines before and after IFNγ treatment (Figure 2 and Figures S2, S3). While basal expression of MHC II was minimal in both KD and Sc cells, IFNγ induced expression of MHC II in control cells that was significantly lower in the KD cells (*p* < 0.05) (Figures 2A,B and Figures S2B, S3A). As described for gynecological cancer cells bearing somatic *JAK1* mutations (17), we also observed lower surface MHC I expression by JAK1-deficient hTERT urothelial cells compared with control cells at baseline. After IFNγ stimulation, however, MHC I was upregulated by both control and KD cells with no significant difference (Figures 2C,D and Figures S2C, S3B). Expression of ICAM-1 was reduced in KD cells at baseline and expressed at significantly lower levels after IFNγ stimulation compared with Sc control (*p* < 0.05; Figures 2E,F and Figures S2D, S3C). In keeping with the effects of other reported somatic mutations associated with IFNγ resistance in cancer cells (28, 29), we observed reduced PD-L1 upregulation after IFNγ stimulation in KD cells compared to Sc (*p* < 0.05; Figures 2G,H and Figures S2E, S3D). Together these data indicate that JAK1 functions to regulate the expression of multiple immunomodulatory cell surface molecules in urothelial cells and that JAK1-deficiency alters urothelial immunomodulatory phenotype.

### JAK1 Deficiency Impairs Apoptosis in Response to IFNγ in hTERT Urothelial Cells

**Figure 3:**
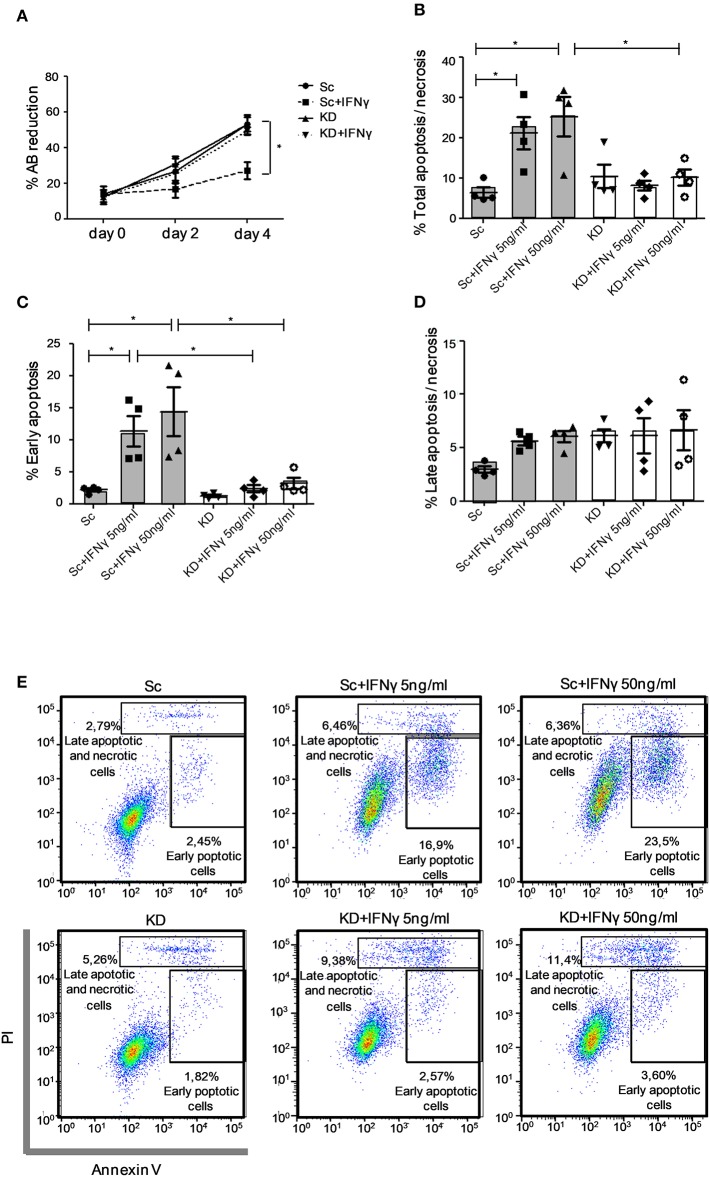
JAK1-deficient hTERT urothelial cells demonstrate preserved population growth and reduced apoptosis in response to IFNγ. **(A)** KD and Sc hTERT immortalized urothelial cell lines were cultured with addition of Alamar Blue (AB) dye and stimulated with IFNγ (5 ng/ml) for the given time points. The capacity of viable cells to reduce AB dye was used as a proxy for cell number. Data **(A)** is from five independent experiments. **(B–E)** KD and Sc hTERT urothelial cell lines were stimulated with the given concentrations of IFNγ for 5 days. Percentage of early and late apoptosis was quantified with Annexin V/PI apoptosis detection kit by flow cytometry. Data **(B–D)** are from five independent experiments, data **(E)** is from a representative experiment. One-way ANOVA with Tukey's Multiple Comparison post-test. ^*^P < 0.05. Error bars represent the SE.

JAK1 KD had no effect on basal growth kinetics of immortalized hTERT urothelial cell lines assessed using an Alamar Blue (AB) reduction assay. In both Sc and KD cell lines, the culture biomass increased steadily over 4 days, indicating a similar increase in cell number over time. IFNγ stimulation significantly inhibited the rise in population in the Sc cell line (*p* < 0.05; Figure 3A), in keeping with an anti-proliferative effect and/or increased cell death. By contrast, growth kinetics of the JAK1-deficient hTERT urothelial cell population showed no significant change following IFNγ treatment (Figure 3A). To further investigate this, we tested whether JAK1-deficient cells are resistant to IFNγ-induced apoptosis using Annexin V/PI staining. In control Sc hTERT urothelial cells, IFNγ induced cell death (Figures 3B,E) which was mainly due to an increase in cells in early apoptosis identified as an Annexin V+/PI dim population (Figures 3C,E), with minimal effect on late apoptosis/necrosis (Figures 3D,E). By contrast, there was no significant impact of IFNγ stimulation on any stage of cell death analyzed in the KD cell line (Figures 3B–E), leading us to conclude that JAK1 is required for IFNγ-mediated apoptosis in urothelial cells. Together, our data support a requirement for JAK1 in regulating urothelial cell survival and homeostasis in response to IFNγ.

### Defective Lymphocyte-Mediated Killing of JAK1-Deficient hTERT Urothelial Cells After IFNγ Stimulation

**Figure 4:**
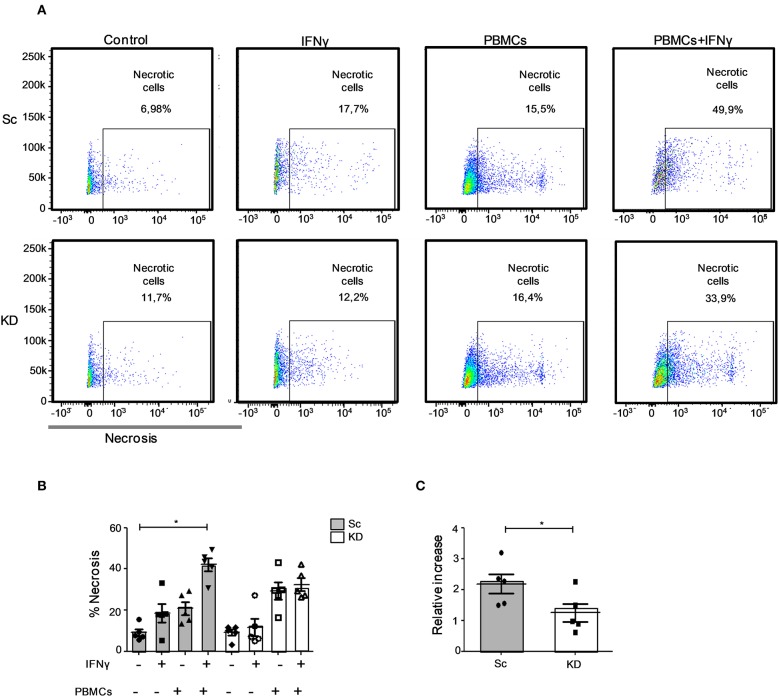
JAK1-deficient hTERT urothelial cells showed reduced lymphocyte-mediated killing in response to IFNγ. JAK1-deficient and Sc hTERT immortalized urothelial cell lines were pretreated or not with IFNγ and cultured overnight with 25 U/ml IL-2 and monocyte-depleted PBMCs (50:1). **(A,B)** Necrosis induction in JAK1-deficient and Sc hTERT immortalized urothelial cell lines. One-way ANOVA with Dunn's multiple comparisons post-test. **(C)** Relative change of necrosis compared to untreated. Two-tailed Mann Whitney *U*-test. Data is from five independent experiments. ^*^P < 0.05. Error bars represent the SE.

We next sought to establish whether the reduced MHC I and ICAM-1 levels observed would impair immune cell recognition of JAK1-deficient hTERT urothelial cells (9, 10, 30, 31). We tested this using a lymphocyte assay where killing can be mediated by NK or CD8+ T cells through multiple mechanisms including shared perforin-granzyme and death receptor/death ligand mechanisms. IFNγ pre-treatment of the Sc hTERT cell line resulted in significant enhancement in urothelial cell lysis by third party, IL-2 expanded primary lymphocytes (measured as necrotic cells, *p* < 0.05; Figures 4A,B). Importantly, lymphocyte mediated cell death was significantly reduced in KD compared to Sc cells following IFNγ pre-treatment (*p* < 0.05; Figure 4C), suggesting that JAK1 deficiency confers resistance to immune cell killing.

### Role of JAK1 in Urothelial Cytodifferentiation

**Figure 5:**
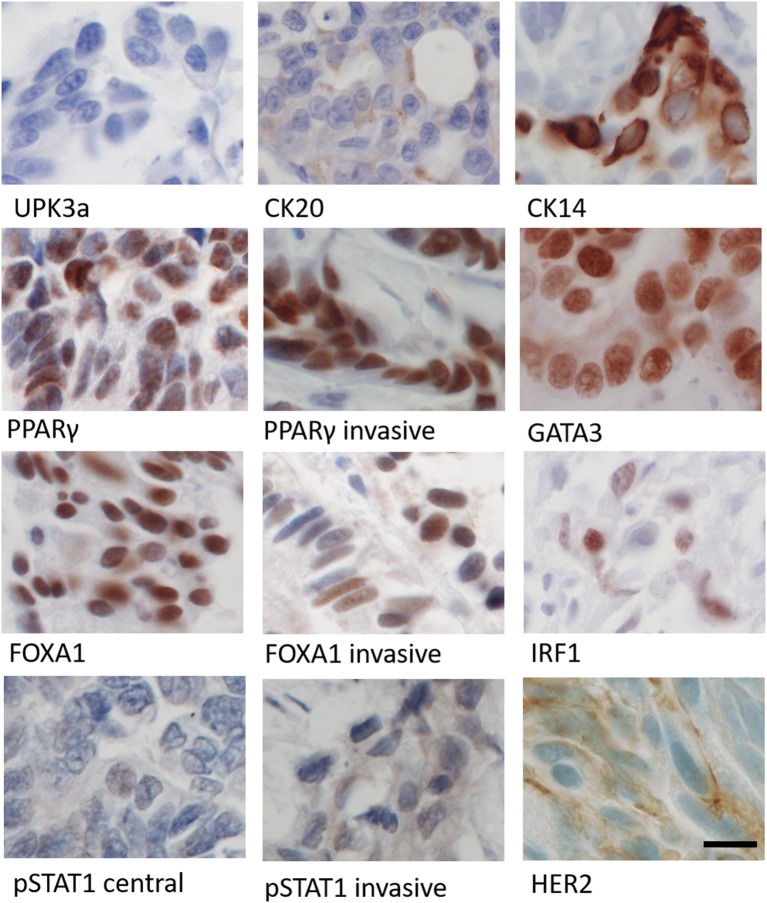
Immunohistochemical labeling of patient's tumor using panel of antibodies against differentiation (UPK3a, CK20, CK14), transcription factor (PPARγ, GATA3, FOXA1, IRF1), and cell signaling (pSTAT1 and HER2) markers. Images from central region of the tumor, except where marked as invasive. Parallel human tissue controls for antibodies are shown in Figure S3. Scale bar = 12 μm.

Histological examination of the invasive urothelial carcinoma found in the JAK1-deficient patient revealed a heterogeneous tumor with distinct central and invasive regions (Figure 5 and Figure S4). The tumor showed no infiltrating immune cells, with only infrequent cells identified in the stroma or vasculature (Figure S5).

Muscle-invasive bladder cancers are classified as “luminal” (urothelial differentiated) or “basal-like” (squamous undifferentiated) subtypes based on signature markers (32). Using antibodies against urothelial differentiation markers, the tumor was negative for uroplakin 3a, whilst cytokeratin (CK) 20 expression was weak and confined to the central region of the tumor. The squamous marker CK14 was present in a small patch of invasive cells but was otherwise absent. Transcriptional regulators of urothelial differentiation PPARγ, GATA3, FOXA1, and IRF1 showed nuclear localization, with weak variable expression of IRF1; pSTAT1 expression was cytoplasmic and weak in both central and invasive regions. Human epidermal growth factor receptor 2 (HER2) expression was variable within the central portion of the tumor, with some areas containing scant membrane and cytoplasmic expression, but negative in other areas, including the invasive front. These findings indicated the tumor to be fundamentally luminal in subtype, albeit with a suppressed urothelial differentiation phenotype. Given the dual role of IRF1 in both IFNγ signaling and PPARγ-mediated urothelial differentiation, this led us to test experimentally whether JAK1 has a role in urothelial cell differentiation.

**Figure 6:**
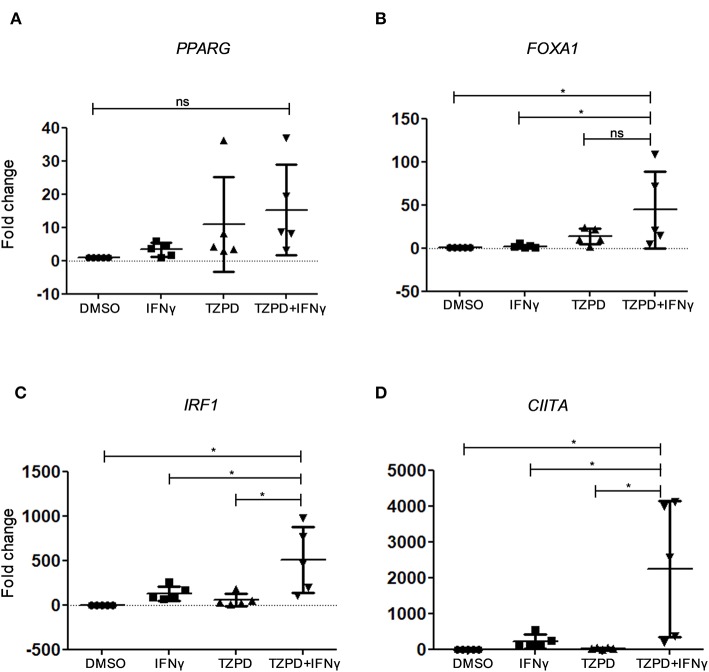
Expression of IFNγ-regulatory genes and intermediary transcription factors in NHU cells by RT-qPCR. **(A–D)** RT-qPCR analysis of gene expression in five non-immortalized NHU cell cultures obtained from five different donors, with or without IFNγ (200 U/ml) and/or TZ (1 μM) + PD153035 (1 μM) stimulation for 48 h compared with the vehicle-only (0.1% DMSO) control. Data are from five independent experiments each using a different NHU cell donor. One-way ANOVA with Tukey's Multiple Comparisons Test. ^*^P < 0.05. Error bars represent the SE.

As the immortalized hTERT urothelial cell line is not suitable for differentiation studies, non-immortalized NHU cell cultures were generated from five individual donors. NHU cells were induced to differentiate by coactivation of PPARγ and inhibition of EGFR signaling, using a combination of troglitazone and PD153035 (TZ/PD) (27). These conditions induce gene expression changes associated with urothelial differentiation via PPARγ-dependent transcription of intermediary transcription factors, including FOXA1 and IRF1 (33). As *IRF1* knock down limits uroplakin expression and *IRF1* induction was impaired in JAK1-deficient cells (Figure 1B), we tested by RT-qPCR whether IFNγ modulates expression of genes associated with IFNγ signaling and urothelial cytodifferentiation pathways. As expected, TZ/PD induced up-regulation of *PPARG* and *FOXA1* (Figures 6A,B). IFNγ alone had a small effect resulting in a weak up-regulation of *IRF1, PPARG*, and the major histocompatibility class II transactivator (*CIITA*) (Figures 6A,C,D). Surprisingly however, stimulation with IFNγ+TZ/PD up-regulated all four genes and substantially increased expression of *IRF1, FOXA1*, and *CIITA* compared with TZ/PD alone (Figures 6A–D). These data suggest that the combination of both IFNγ and TZ/PD significantly increases expression of transcription factors involved in urothelial cytodifferentiation and supports a previously unknown role for IFNγ in modulating urothelial phenotype.

## Discussion

The fundamental importance of JAK1 signaling has been highlighted by perinatal lethality of JAK1 knock-out mice (3) and, in keeping with this, germline mutations causing complete loss of JAK1 function have not been described in humans. We recently reported the first case of germline loss of function mutations causing partial JAK1 deficiency in humans which resulted in immunodeficiency characterized by mycobacterial infection, suggesting a dominant effect on the IFNγ pathway, and high-grade bladder carcinoma (1). The early-onset and aggressive nature of the malignancy, along with the fact that *JAK1* is a hotspot for damaging somatic mutations in bladder carcinoma (28), led us to investigate whether impaired JAK1 function may be a specific predisposing factor for urothelial carcinoma. The data presented here demonstrate that JAK1 is important for multiple aspects of urothelial cell biology and highlight mechanisms by which loss of JAK1 function may promote tumorigenesis in this cell type.

Using a JAK1-deficient urothelial cell line model, we show that loss of JAK1 function impaired induction of apoptosis in response to IFNγ suggesting a role for JAK1 in regulating intrinsic urothelial cell homeostasis. In addition, JAK1-deficient urothelial cell lines demonstrated reduced surface expression of ICAM-1 following IFNγ stimulation, which was associated with resistance to lymphocyte-mediated cell lysis that is known to correlate with cell surface expression of this molecule (10, 34). While we did not observe a significant effect of JAK1 deficiency on IFNγ-mediated upregulation of MHC I in our model, JAK1 was shown to be required for expression of MHC II that can mediate tumor and self-antigen presentation in non-professional antigen presenting cells (35). This difference could be explained by the fact that MHC I is highly expressed constitutively on urothelial cells whereas MHC II expression, like ICAM-1, is induced *de novo* by IFNγ activity (Figure 2) and hence demonstrates a highly specific directed response.

Together these data suggest that JAK1-deficient urothelial cells are less susceptible to IFNγ-mediated apoptosis, immune cell recognition and immune-mediated cell death. The absence of an immune infiltrate observed in the patient's tumor by immunohistochemistry supports our conclusion that loss of JAK1 in bladder cancer cells results in a poorly immunogenic tumor. IFNγ is of specific relevance in bladder cancer as it is a major cytokine released by tumor infiltrating lymphocytes thought to be important for anti-tumor responses and has potential for use as a biomarker of outcome in this condition (36–39). It has already been reported that damaging mutations in the IFNγ signaling pathway and antigen presentation pathway are associated with metastasis and higher resistance to the checkpoint blocking therapy with anti-PD-L1/PD-1 in a number of tumor types, including bladder cancer (29, 30, 40). Our data suggest loss of function *JAK1* mutations are a risk factor for lower tumor cell PD-L1 expression which could impair responsiveness to anti-PD-1 therapy used for advanced urothelial carcinoma (41). Further correlation of tumor genetics with clinical response to treatment is required to test this in practice.

In addition to impacting immune-related functions, we observed a potential role for JAK1 and IFNγ signaling in urothelial cell differentiation. It has been shown that NHU cells can be induced to differentiate using PPARγ ligands and concurrent EGFR inhibition, for example using TZ/PD (27). In this study, we show that IFNγ had a significant effect on the induction of the transcription factors IRF1 and FOXA1, both known to be involved in urothelial cell differentiation induced by PPARγ activation (33). In particular, IRF1 is a common downstream mediator for PPARγ and IFNγ signaling pathways influencing both urothelial differentiated phenotype and immune cell interactions. This suggests that IRF1 may be a potential novel target for modulating immunotherapy outcomes in urothelial cancer. Further research is needed to understand the mechanisms of IFNγ interaction with PPARγ and EGFR signaling in urothelial cell differentiation and tumorigenesis.

In summary our findings highlight previously unknown roles for JAK1 in urothelial cell immune recognition and differentiation. Our data suggest that loss of JAK1 function through germline or somatic mutation promotes malignant transformation of urothelial cells, which are intrinsically less immunogenic.

Our results add further weight to arguments for sequencing urothelial cell tumors for clinical trials of immunotherapy agents to test whether the mutational burden of JAK1 and other IFNγ-related genes represent a biomarker for responsiveness to treatment in bladder cancer, which can more accurately predict the clinical outcome of these patients.

## Ethics Statement

Blood samples and biopsies were obtained with ethical approval (National Research ethics numbers 08/H0720/46, 99/095 and 02/208) and informed consent from all subjects in accordance with the Declaration of Helsinki.

## Author Contributions

VD-C, JS, and SB conceived, designed and coordinated the study. VD-C, JP, and JH performed the experiments. SB, JS, and AT provided clinical data and gave critical advice. VD-C, JP, JH, AA, JS, and SB analyzed the data and interpreted the results. AM performed bioinformatic analysis. VD-C and SB wrote the manuscript. VM, AA, JS, and AT gave administrative, technical, or material support. The final version of the manuscript was reviewed by all the coauthors.

### Conflict of Interest Statement

The authors declare that the research was conducted in the absence of any commercial or financial relationships that could be construed as a potential conflict of interest.
