# Supplementary material for: Loss of Janus Associated Kinase 1 Alters Urothelial Cell Function and Facilitates the Development of Bladder Cancer
Source: Front Immunol. 2019 Sep 10;10:2065. doi: 10.3389/fimmu.2019.02065 (PMC6746825; doi:10.3389/fimmu.2019.02065)
Supplement: Supplementary file 1 [file Data_Sheet_1.doc]

**Supplementary methods**

**Patients and human cell lines**

Patient and control skin fibroblasts as well as 293T cells were cultured in DMEM medium and 10% heat-inactivated FCS and 1% P/S. For the different experiments fibroblasts were detached using Accutase® solution (A6964, Sigma Aldrich). Regular checks for *Mycoplasma spp* contamination were performed. Informed written consent was obtained in accordance with the Declaration of Helsinki and ethical approval from the Great Ormond Street Hospital for Children NHS Foundation Trust and the Institute of Child Health Research Ethics Committee (Reference Number: 06/Q0508/16).

**Immunohistology**

5µM formalin-fixed tissue sections were dewaxed in xylene, then rehydrated through ethanol into water. Details of the antibodies used and antigen retrieval steps are given in Supplementary Table S3. For FOXA1, UPK3a, CK14 and CK20, endogenous avidin and biotin were blocked and antigen retrieval performed. After 16h incubation of primary antibody at 4°C, slides were washed, incubated in biotinylated secondary antibody and visualized by addition of streptavidin-biotin horseradish peroxidase complex (DAKO) and 3,3’-diaminobenzidine (Sigma Aldrich). For PPARγ, IRF1 and GATA3, antigen retrieval was performed before incubating with primary antibody for 16h at 4°C. Visualization of bound antibody was performed using an ImPRESS™ Excel Polymer system (Vecor labs), according to manufacturer’s instructions. HER2 labelling was performed in a BenchMark Ultra (Ventana) automated staining system according to manufacturer’s instructions. Epitope retrieval was performed for 36 minutes using Cell Conditioning 1 solution (Ventana) followed by incubation for 60 minutes with HER2 primary antibody. Visualization of antigenic sites was performed using an UltraView Universal DAB detection kit (Ventana). Immunocyte markers CD3, CD4, CD8, CD56 and CD57 were labelled using an automated Dako platform. All slides were counterstained in Mayer’s haematoxylin and mounted in DPX (Sigma).

**Table S1. Primers used for RT-qPCR and RT-PCR**

| **Target** | **Forward (5’ 🡪 3’)** | **Reverse (5’ 🡪 3’)** |
| --- | --- | --- |
| JAK1 | TGGATCTCTTCATGCACCGGA | ATGAATGGGCCACACTCACTG |
| IRF1 | CAGAGAAAAGAAAGAAAGT | CATCAGAGAAGGTATCAG |
| GAPDH | GAGCCACATCGCTCAGACAC | CATGTAGTTGAGGTCAATGAAGG |
| FOXA1 | CAAGAGTTGCTTGACCGAAAGTT | TGTTCCCAGGGCCATCTGT |
| PPARγ | GAACAGATCCAGTGGTTGCAG | CAGGCTCCACTTTGATTGCAC |
| CIITA | AGGGACCCATCCAGTTTGTC | TGGCCGGTCTGGAGATGTTG |

RT-qPCR= Real time quantitative polymerase chain reaction, RT-PCR= Reverse transcription polymerase chain reaction, JAK1= Janus associated kinase 1, IRF1= Interferon regulatory factor 1, GAPDH= glyceraldehyde-3-phosphate dehydrogenase, FOXA1= Forkhead box protein A1, PPARγ= Peroxisome proliferator-activated receptor gamma, CIITA= class II, major histocompatibility complex, transactivator.

**Table S2: Antibodies used for flow cytometry**

| **Antibody** | **Catalog No.** | **Supplier** |
| --- | --- | --- |
| anti-HLA-ABC (FITC) | IM1838U | Beckman Coulter Immunotech |
| anti-CD119 (IFNGR1) (PE) | 558937 | BD Bioscience |
| anti-CD54 (ICAM-1) (APC) | 559771 | BD Bioscience |
| anti-CD274 (PD-L1) (PE) | 329705 | BioLegend |
| anti-HLA-DR (PerCP) | 347402 | BD Bioscience |
| Anti-STAT1 (pY701) (Alexa Fluor 467) | 612557 | BD Bioscience |

# HLA= Human leukocyte antigen, IFNGR= Interferon gamma receptor, ICAM-1= Intracellular adhesion molecule 1 (CD54), PD-L1= Programmed death-ligand 1, STAT1= Signal transducer and activator of transcription, FITC= Fluorescein-5-isothiocyanate, PE= Phycoerythrin, APC= Allophycocyanin, PerCP= Peridinin-Chlorophyll-protein.

**Table S3. Primary antibodies used for immunohistochemistry**

| **Antibody target** | **Clone or name** | **Host species** | **Concentration** | **Antigen retrieval** | **Supplier** |
| --- | --- | --- | --- | --- | --- |
| UPK3a | AU1 | Mouse mAb | 0.25 µg/mL | Boil 10 min 10 mM citric acid pH 6.0 | ProgenBiotechnik |
| CK14 | LL002 | Mouse mAb | 0.2 µg/mL | 0.1% trypsin at 37^o^C 1 min then boil 10 min 10 mM citric acid pH 6.0 | Biorad |
| CK20 | Ks20.8 | Mouse mAb | 0.1 µg/mL | 0.1% trypsin at 37^o^C 10 min | Leica Biosystems |
| GATA3 | D13C9 | Rabbit mAb | 1:800 | boil 10 min 10 mM citric acid pH 6.0 | Cell Signalling Technology |
| pSTAT1 (Tyr701) | 58D6 | Rabbit mAb | 1:8000 | boil 10 min in 10mM Tris-EDTA pH 9.0 | Cell Signalling Technology |
| HER2/NEU | 4B5 | Rabbit monoclonal | As supplied | 36 min in Cell Conditioning 1 solution (Ventana) | Ventana |
| PPARγ | E8 | Mouse mAb | 0.1 µg/mL | boil 10 min 10 mM citric acid pH 6.0 | Santa Cruz Biotechnology |
| FOXA1 | Q6 | Mouse mAb | 0.4 µg/mL | boil 10 min 10 mM citric acid pH 6.0 | Santa Cruz Biotechnology |
| IRF-1 | D5E4 | Rabbit mAb | 1:300 | boil 10 min 10 mM citric acid pH 6.0 | Cell Signalling Technology |
| CD3: Dako | N/A | Rabbit antiserum | As provided | Dako Omnis platform | Dako |
| CD4 | 4B12 | Mouse mAb | As provided | Dako Omnis platform | Dako |
| CD8 | C8/144B | Mouse mAb | As provided | Dako Omnis platform | Dako |
| CD56 | 123C3 | Mouse mAb | As provided | Dako Omnis platform | Dako |
| CD57 | TB01 | Mouse mAb | As provided | Dako Omnis platform | Dako |

UPK3a= Uroplakin 3a, CK= Cytokeratin, STAT1= Signal transducer and activator of transcription, HER2 human epidermal growth factor receptor 2, PPARγ = Peroxisome proliferator-activated receptor gamma, FOXA1 = Forkhead box protein A1, IRF1= Interferon regulatory factor 1.

**
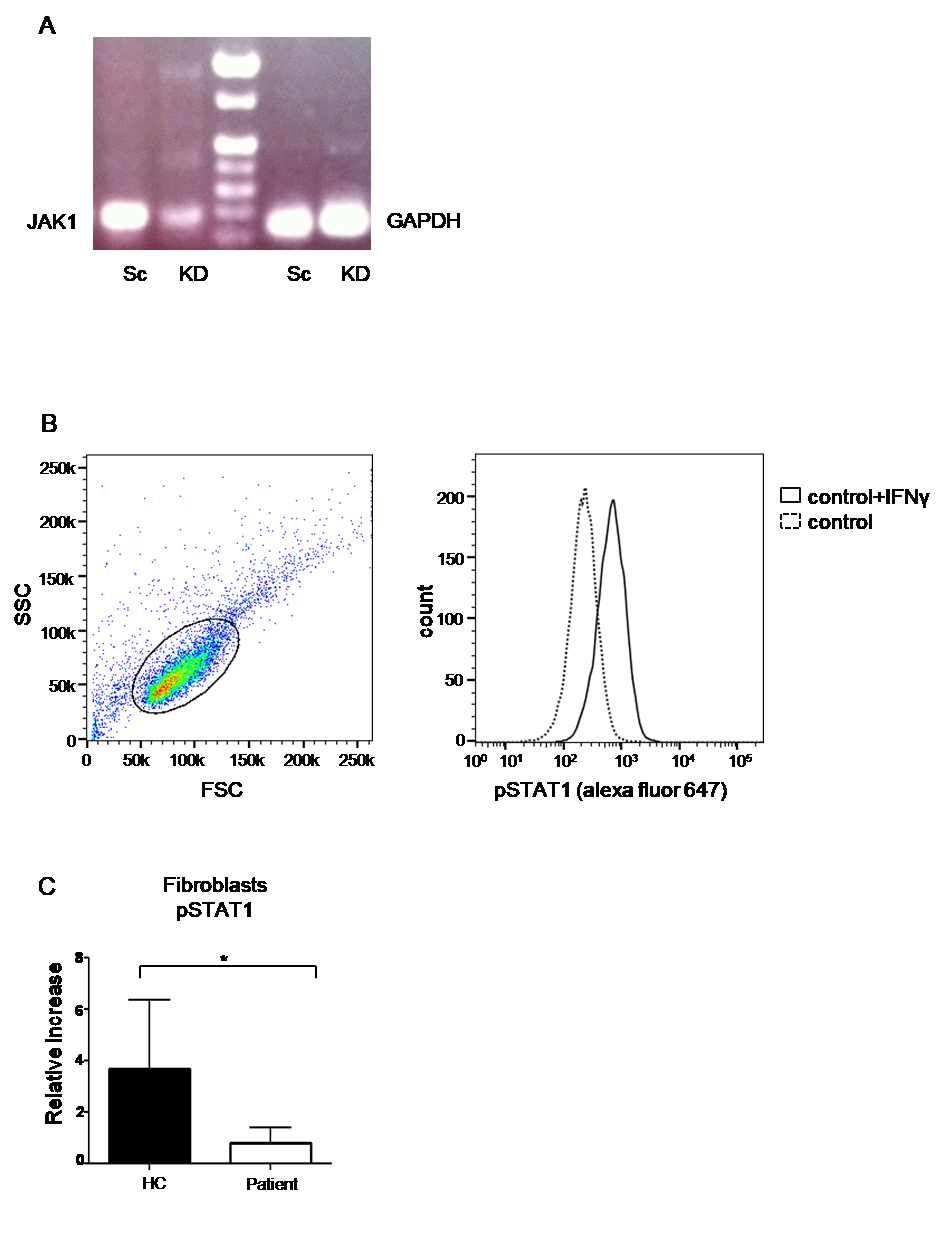
**

**Supplementary Figure S1.**

**(A) *JAK1* expression in JAK1-deficient hTERT urothelial cells and control.** hTERT urothelial cells were transfected with vectors expressing JAK1 shRNA and scrambled control shRNA. RT-PCR analysis of *JAK1* expression is shown. Data is representative of two independent experiments.

**(B) Gating strategy for analysis of STAT1 phosphorylation and IFNGR expression by flow cytometry**

(left) SSC vs FCS density plot: a gate has been applied to identify the urothelial cell population excluding debris. (right) Cells within the gate defined are represented in a histogram to evaluate the relative expression of a marker; pSTAT1 shown here as an example.

**(C) Impaired STAT1 phosphorylation in fibroblasts of the patient with JAK1 deficiency**

Analysis of JAK/STAT signalling by flow cytometry in control and JAK1 deficient fibroblasts after stimulation with IFNγ (100ng/ml). Data are from three independent experiments. Two-tailed Mann Whitney test. *P <0.05. Error bars represent the SE.


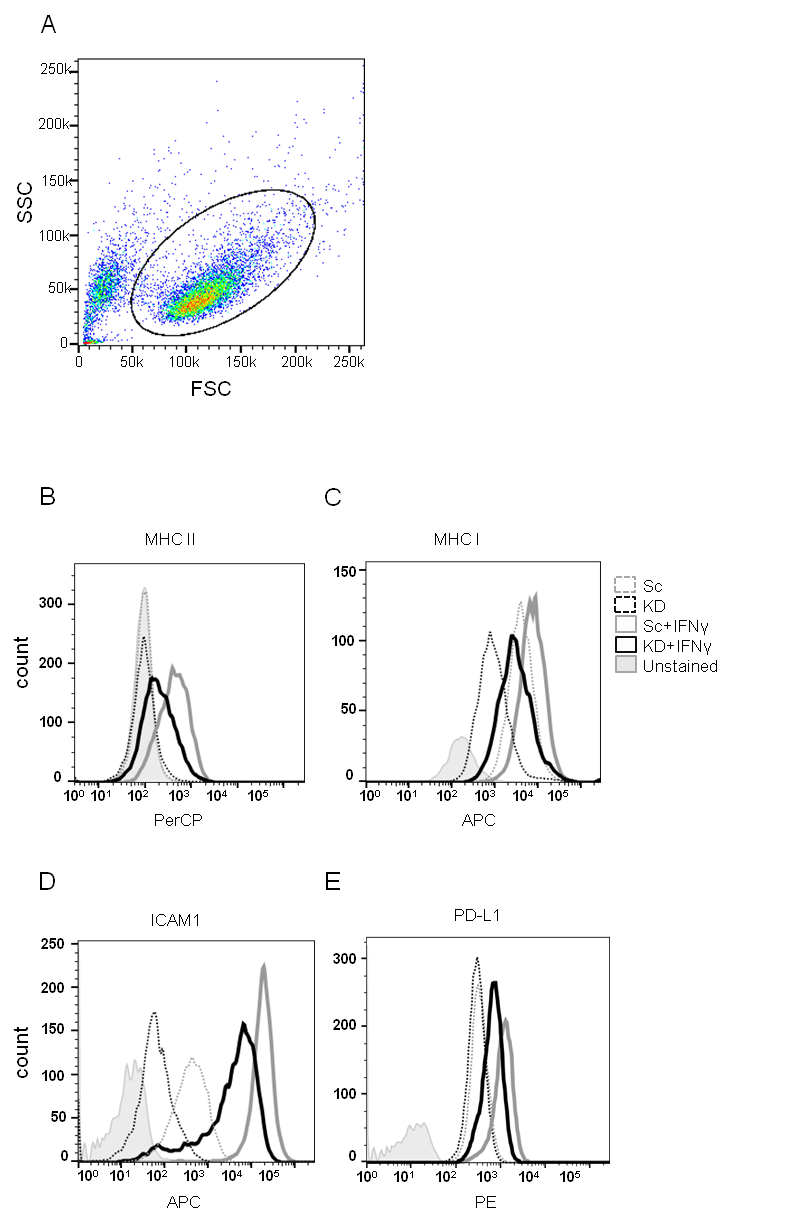


**Supplementary Figure S2.**

**(A) Gating strategy for the identification of surface markers by flow cytometry**

SSC vs FCS density plot: a gate has been applied to identify the urothelial cell population excluding debris.

**(B-E)** **Analysis of MHC I/II, ICAM-1 and PD-L1 expression in hTERT urothelial cells by flow cytometry.** Cells within the gate defined in (A) are represented in a histogram to evaluate the relative expression of a marker. Overlay of a negative unstained population onto the stained population allows easy identification of the positive cells. KD and Sc hTERT urothelial cell lines were stimulated with IFNγ and MHC I/II, ICAM-1 and PD-L1 expression analyzed by flow cytometry. Data are from a representative experiment.

**
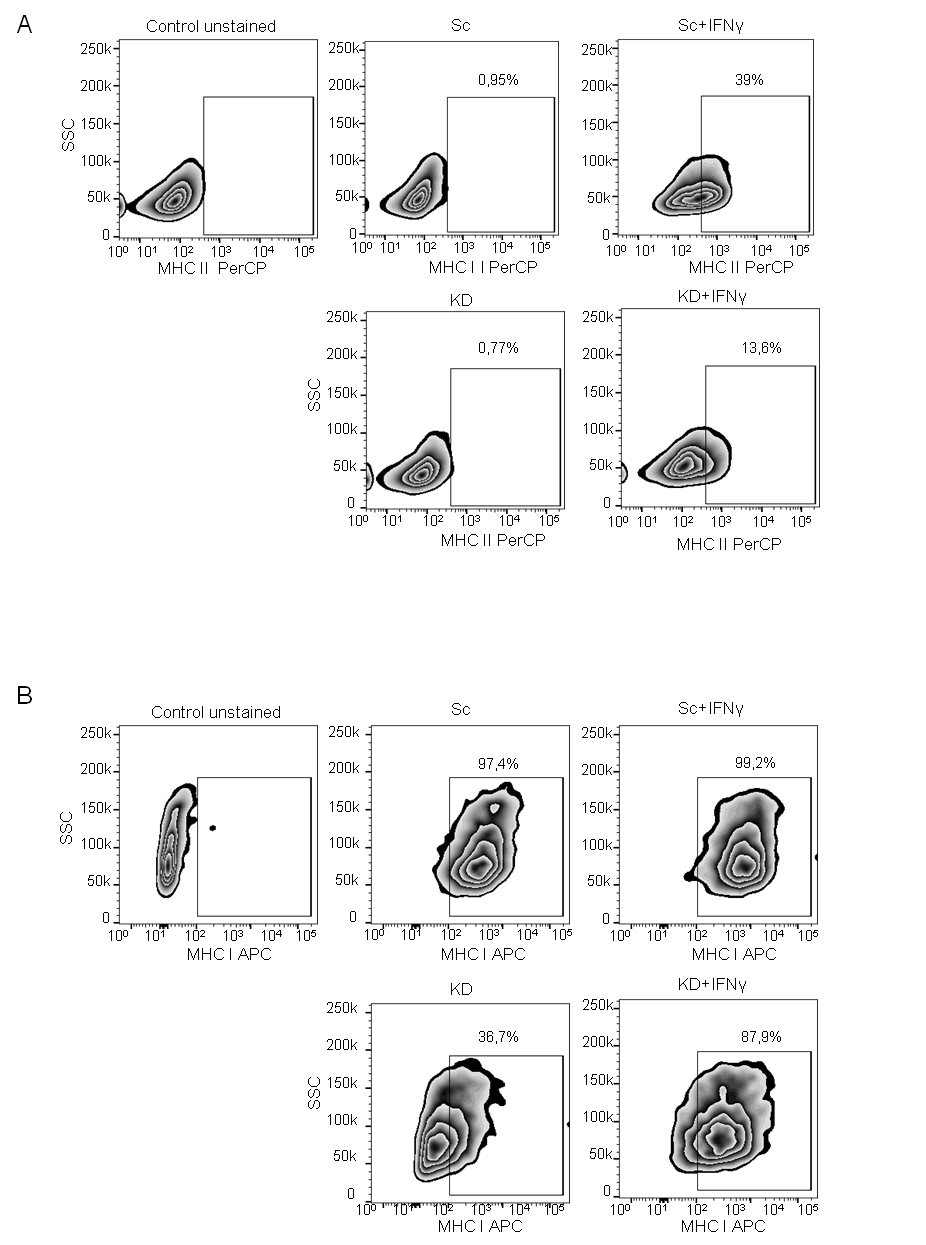
**

**
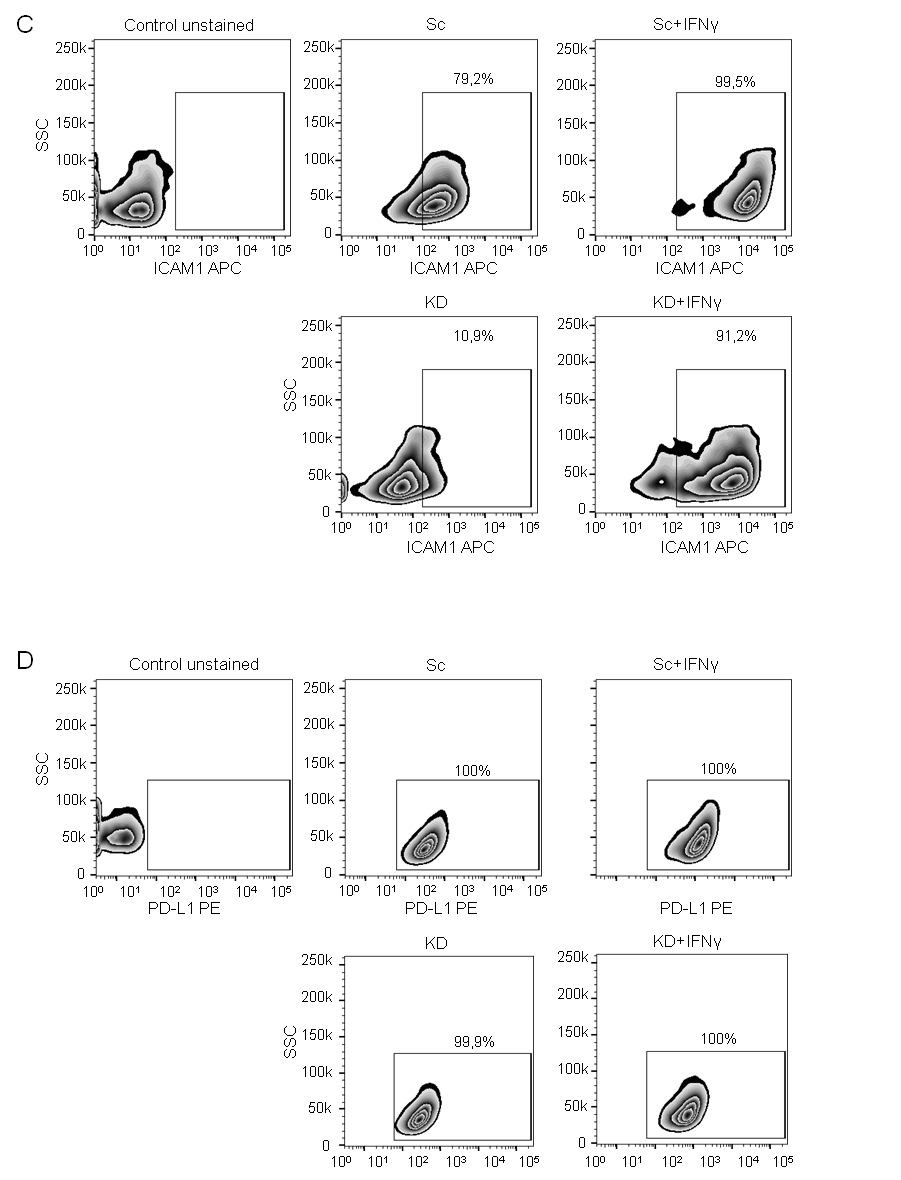
**

**Supplementary Figure S3.**

**Percentage of hTERT urothelial cells expressing MHC II, MHC I, ICAM-1 and PD-L1 measured by Flow Cytometry.**

Flow cytometry analysis of surface receptor expression in KD and Sc hTERT urothelial cell lines before and after IFNγ stimulation is shown. The urothelial cell population was initially gated as shown in Supplementary Figure S2a to exclude debris. A negative unstained population was used to set subsequent gates to allow identification of the positive (stained) cells. Percentages of positive cells are shown for **(A)** MHC II **(B)** MHC I **(C)** ICAM1 and **(D)** PD-L1 expression. Data are from a representative experiment.

**
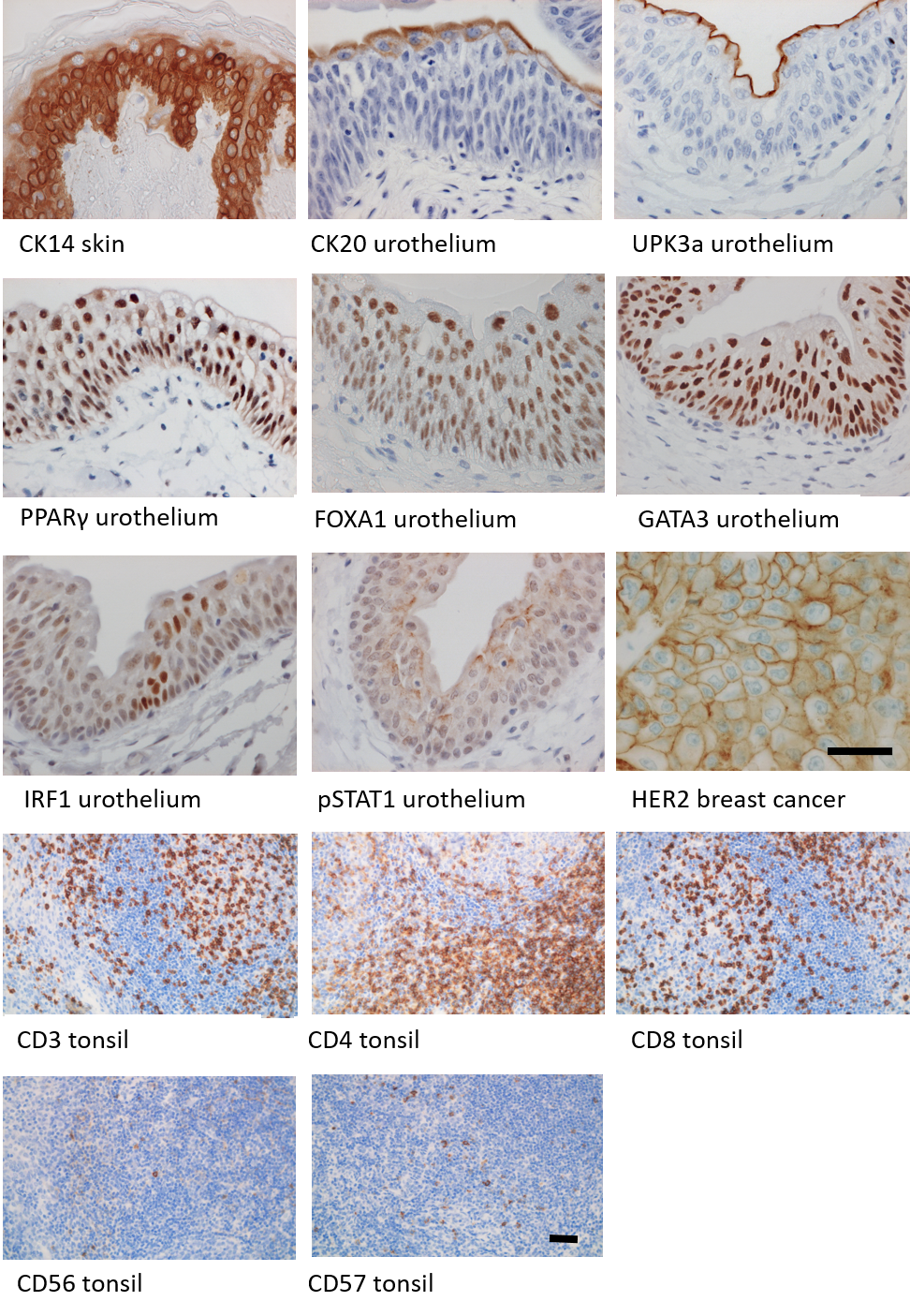
**

**Supplementary Figure S4.**

**Positive control tissues for antibodies labelled in parallel to IHC shown in Figure 5.** Scale bar top panel = 50 μm; bottom panel = 100μm. Data are from a representative experiment.

**
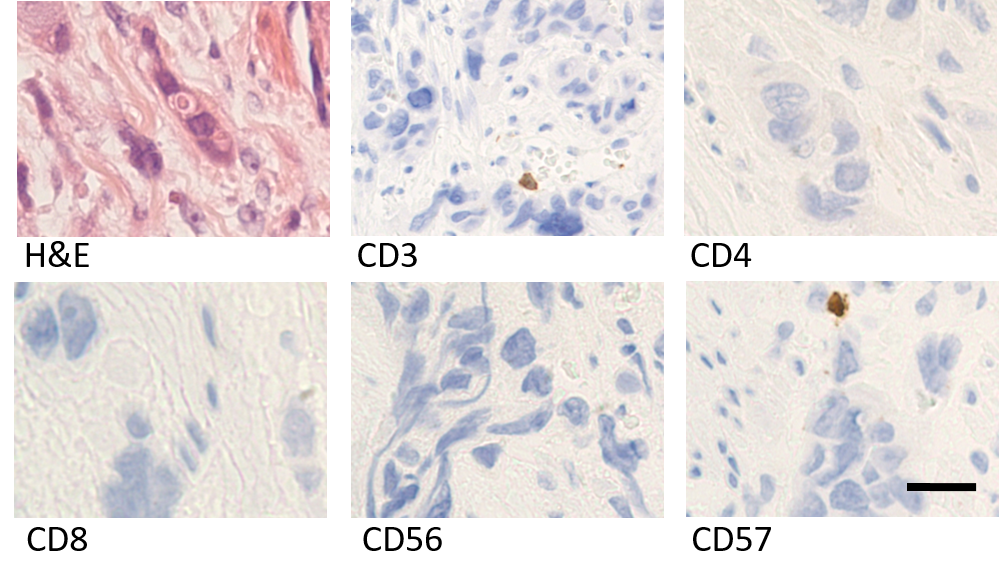
**

**Supplementary Figure S5.**

**Immunohistochemistry for immune cell markers in invasive portion of tumour showing dearth of tumour-infiltrating immune cells.**

Human tonsil controls for antibodies are included in Figure S3. Scale bar = 50μm. Data are from a representative experiment.
